# Minor Contribution of Endogenous GLP-1 and GLP-2 to Postprandial Lipemia in Obese Men

**DOI:** 10.1371/journal.pone.0145890

**Published:** 2016-01-11

**Authors:** Niina Matikainen, Elias Björnson, Sanni Söderlund, Christofer Borén, Björn Eliasson, Kirsi H. Pietiläinen, Leonie H. Bogl, Antti Hakkarainen, Nina Lundbom, Angela Rivellese, Gabriele Riccardi, Jean-Pierre Després, Natalie Alméras, Jens Juul Holst, Carolyn F. Deacon, Jan Borén, Marja-Riitta Taskinen

**Affiliations:** 1 Research programs Unit, Diabetes and Obesity, University of Helsinki and Heart and Lung Center, Helsinki University Hospital, Helsinki, Finland; 2 Endocrinology, Abdominal Center, Helsinki University Hospital, Helsinki, Finland; 3 Department of Molecular and Clinical Medicine/Wallenberg Laboratory, University of Gothenburg and Sahlgrenska University Hospital, Gothenburg, Sweden; 4 Department of Radiology, HUS Medical Imaging Center, Helsinki University Central Hospital, University of Helsinki, Helsinki, Finland; 5 Department of Clinical Medicine and Surgery, Federico II University, Naples, Italy; 6 Institut Universitaire de Cardiologie et de Pneumologie de Québec, Québec City, Québec, Canada; 7 NNF Centre for Basic Metabolic Research, and Department of Biomedical Sciences, Faculty of Health and Medical Sciences, University of Copenhagen, Copenhagen, Denmark; University of Bremen, GERMANY

## Abstract

**Context:**

Glucose and lipids stimulate the gut-hormones glucagon-like peptide (GLP)-1, GLP-2 and glucose-dependent insulinotropic polypeptide (GIP) but the effect of these on human postprandial lipid metabolism is not fully clarified.

**Objective:**

To explore the responses of GLP-1, GLP-2 and GIP after a fat-rich meal compared to the same responses after an oral glucose tolerance test (OGTT) and to investigate possible relationships between incretin response and triglyceride-rich lipoprotein (TRL) response to a fat-rich meal.

**Design:**

Glucose, insulin, GLP-1, GLP-2 and GIP were measured after an OGTT and after a fat-rich meal in 65 healthy obese (BMI 26.5–40.2 kg/m^2^) male subjects. Triglycerides (TG), apoB48 and apoB100 in TG-rich lipoproteins (chylomicrons, VLDL_1_ and VLDL_2_) were measured after the fat-rich meal.

**Main Outcome Measures:**

Postprandial responses (area under the curve, AUC) for glucose, insulin, GLP-1, GLP-2, GIP in plasma, and TG, apoB48 and apoB100 in plasma and TG-rich lipoproteins.

**Results:**

The GLP-1, GLP-2 and GIP responses after the fat-rich meal and after the OGTT correlated strongly (r = 0.73, *p*<0.0001; r = 0.46, *p*<0.001 and r = 0.69, *p*<0.001, respectively). Glucose and insulin AUCs were lower, but the AUCs for GLP-1, GLP-2 and GIP were significantly higher after the fat-rich meal than after the OGTT. The peak value for all hormones appeared at 120 minutes after the fat-rich meal, compared to 30 minutes after the OGTT. After the fat-rich meal, the AUCs for GLP-1, GLP-2 and GIP correlated significantly with plasma TG- and apoB48 AUCs but the contribution was very modest.

**Conclusions:**

In obese males, GLP-1, GLP-2 and GIP responses to a fat-rich meal are greater than following an OGTT. However, the most important explanatory variable for postprandial TG excursion was fasting triglycerides. The contribution of endogenous GLP-1, GLP-2 and GIP to explaining the variance in postprandial TG excursion was minor.

## Introduction

Enteroendocrine cells in the gastrointestinal tract may act as nutritional sensors because they secrete a variety of peptide hormones, which regulate energy balance, satiety and metabolism in response to oral nutrient ingestion. These hormones include glucagon-like peptide (GLP)-1 secreted by L-cells in distal small intestine and glucose-dependent insulinotropic polypeptide (GIP) secreted by K-cells [1]. Both peptides stimulate insulin secretion, and together they are responsible for the incretin effect, *i*.*e*., the augmentation of insulin secretion observed when glucose is administered orally rather than intravenously [2, 3]. Although they act similarly to stimulate insulin secretion via specific receptors expressed by the β-cell, they differentially influence glucagon secretion from the α-cells [1], with GLP-1 inhibiting and GIP stimulating secretion.

Like GLP-1, GLP-2 is a product of preproglucagon and is secreted in parallel and in equimolar amounts with GLP-1 from the L-cells in response to carbohydrate and lipid ingestion. However, unlike GLP-1, GLP-2 does not stimulate insulin secretion, but increases glucagon secretion [4, 5], while having a powerful stimulating effect on intestinal growth, mucosal integrity and nutrient absorption. An appetite regulating effect through central nervous system GLP-2 receptors [5, 6] has also been proposed. These properties provide the background for the therapeutic use of degradation resistant GLP-2 in small bowel syndrome and other intestinal malabsorption states [5], but the physiological roles of GLP-2 in humans is far from fully understood.

Defects in both secretion and insulinotropic actions of GLP-1 and GIP appear as early signs of insulin resistance characterizing the metabolic syndrome [7, 8]. Another early and central metabolic defect in the metabolic syndrome is the overproduction of both hepatic VLDL and intestinal chylomicron particles because of an impaired suppressive action of insulin. Such abnormalities lead to the development of an atherogenic dyslipidemia, *i*.*e*., fasting and postprandial hypertriglyceridemia, low high-density lipoprotein (HDL) cholesterol levels and high levels of small low-density lipoprotein (LDL) particles [9–11]. Therefore, even before development of hyperglycemia, increased levels of both TG-rich lipoprotein (TRL) remnants and small dense LDL particles contribute to accelerated atherosclerosis [12].

Interestingly, GLP-1, GLP-2 and GIP have all been implicated in lipid homeostasis. In rodent models, GLP-1 decreases apolipoprotein (apo) B48 production in the intestine [13]. Indirect evidence derived from the pharmacological use of GLP-1 mimetics or dipeptidyl peptidase (DPP) 4-inhibitors strongly suggests that GLP-1, and possibly GIP, reduce intestinal and, in some studies, also hepatic postprandial TRL particles [14, 15]. A kinetic study by Xiao *et al*. [16] also demonstrated inhibition of apoB48 production by Exenatide, a GLP-1 receptor agonist. Interestingly, GLP-2 may have an opposite role by promoting intestinal lipid absorption via induction of CD36/fat acid translocase, as well as by promoting packaging and secretion of apoB48-containing chylomicrons in rodents [17]. In humans, much less is known about the combined physiological effects of the intestinal hormones on regulation of postprandial lipid metabolism, although pharmacological doses of GLP-2 injected subcutaneously in humans were recently reported to rapidly increase apoB48 and TG in TRL, as well as TG levels in plasma during constant intraduodenal feeding [18].

Here we report the endogenous responses of GLP-1, GLP-2 and GIP following a fat-rich mixed meal test and after a standard oral glucose tolerance test (OGTT) in male overweight subjects with a wide range of fasting TG levels. The purpose of this study was to compare the magnitude and time sequence of GLP-1, GLP-2 and GIP responses elicited by a fat-rich mixed meal test to explore the possible concerted action of these intestinal hormones in the regulation of postprandial lipid metabolism.

## Materials and Methods

### Study subjects

We studied 65 overweight healthy men from our Fructose intervention protocol (Clinical Trials NCT01445730) carried out in Helsinki, Naples and Gothenburg. The subjects were recruited via a newspaper advertisement. Males with large waist (>94 cm) and body mass index (BMI) between 26.5 and 40 kg/m^2^ who had been weight stable for at least the last 3 months were included in the study. Each subject underwent a physical examination and laboratory tests to exclude cardiovascular disease, uncontrolled hypertension, type 2 diabetes, hepatic diseases, renal, gastroenteral, thyroid or haematological abnormalities. Subjects with regular daily alcohol consumption (greater than two drink equivalents per day) (*i*.*e*., 20 g pure alcohol) were excluded from the study. None of the subjects used medication or hormones affecting lipoprotein metabolism. The study design was approved by the local Ethics committees at University of Helsinki, Finland, University of Gothenburg, Sweden and University of Naples, Italy and each subject gave written informed consent before participation in the study. All the samples were collected in accordance with the Declaration of Helsinki.

Each subject underwent an oral fat tolerance test, served as a fat-rich mixed meal (in the text referred as a “fat-rich meal”), and a standard OGTT on separate days within 2 weeks. The participants abstained from alcohol and physical exercise for two days before each examination. Each subject was asked to keep a 3-day food diary to ensure that they followed an ordinary isocaloric diet during the study.

After a 12-h fast, subjects received a fat-rich meal consisting of bread, butter, cheese, ham, boiled eggs, fresh red pepper, low fat (1%) milk, orange juice and tea or coffee (63 g carbohydrates, 56 g fat and 40 g protein). The fat-rich meal contained 927 kcal. Blood samples were drawn before and 30, 60, 120, 180 and 240 minutes after the meal for determination of glucose, insulin, GLP-1, GLP-2 and GIP as well as for determination of lipid and lipoproteins. During the test, only water was allowed *ad libitum* and the subjects remained physically inactive.

In addition, a 75 g OGTT was performed after an overnight fast. The subjects consumed 75 g of glucose and blood sampling was done before and 5, 10, 30, 60, 90, 120, 180 and 240 minutes after the OGTT for determination of glucose, insulin, GLP-1, GLP-2 and GIP.

### Biochemical analysis

Fasting and postprandial concentrations of glucose (hexokinase method, Roche Diagnostic Gluco-quant, Mannheim, Germany) and insulin (electrochemiluminescence with Roche sandwich immunoassay on a Cobas autoanalyzer) were measured after the fat-rich meal and the OGTT. Adiponectin was measured by an enzyme-linked immunosorbent assay (R&D Systems, Inc., Minneapolis, USA) [19]. GIP, GLP-1 and GLP-2 plasma concentrations were measured after ethanol extraction (70% vol/vol, final concentration), as described. GIP and GLP-1 were measured using C-terminally directed assays, which detect both the intact peptide and the primary (N-terminally truncated) metabolite; such assays therefore can be designated as “total”, and can be used to reflect the rate of secretion [20]. For GIP [21], we used antiserum code # 80867, which reacts fully with GIP 3–42, but not with GIP 8000, whose chemical nature and relationship to GIP secretion is uncertain. Plasma concentrations of GLP-1 were measured [22] using antiserum code no. 89390, which is specific for the amidated C-terminus of GLP-1. This antiserum reacts fully with GLP-1 9-36amide (and with the small amounts of pancreatic GLP-1 1-36amide), but does not cross-react with major proglucagon fragment from the pancreas. For both assays sensitivity was below 1 pmol/l and intrassay coefficient of variation below 6% at 20 pmol/l. GLP-2 concentrations were measured employing antiserum code no. 92160 [23]. The antiserum is directed against the N-terminus of GLP-2 and therefore measures only fully processed GLP-2 of intestinal origin. Sensitivity for the assay is below 2 pmol/l, and intra-assay coefficient of variation below 6%.

### Isolation of lipoproteins

Lipoprotein fractions [chylomicrons (S_f_>400), large VLDL_1_ particles (S_f_60–400) and smaller VLDL_2_ particles (S_f_20–60)] were separated by density gradient ultracentrifugation. All of these fractions contain both apoB48 and apoB100 particles that were further analyzed using SDS-PAGE, as previously described [24]. TG and cholesterol concentrations in total plasma and lipoprotein fractions were analyzed by automated enzymatic methods using the Konelab 60i analyzer (Thermo Electron Corporation, Vantaa, Finland. Fasting and postprandial apoB48 levels (Elisa kit, Shibayagi Co. Ltd, Shibukawa, Gunma, Japan) were measured in plasma.

### Statistical analyses and calculations

Statistical analyses were performed using GraphPad Prism 6 for Windows (GraphPad Software, Inc., San Diego, CA). Data are presented as mean ± SEM, SD or range. HOMA-IR and HOMA-IS indices were calculated as described previously [25]. Skewed data were either transformed to natural logarithms when indicated, or analyzed using nonparametric tests. The area under curve (AUC) and incremental AUC (*i*.*e*., the AUC above the fasting concentration) for postprandial variables were calculated according to the trapezoid rule. Only AUCs are shown to simplify the results, because fasting values for glucose, insulin, and incretins were similar before fat-rich meal and OGTT. Correlations were calculated using Spearman´s rank test. A *p* value < 0.05 was considered statistically significant. Relative importance analysis was performed in order to estimate how much the different measured variables (including GLP-1, GLP-2 and GIP) contributed to explaining the variance in the parameters of interest (in this case TG plasma AUC, apoB48 AUC and TG chylomicron AUC). If all variables were uncorrelated, this quantification would be equivalent to the variable’s individual R-squared value. However, since this was not the case we used the method for relative importance analysis proposed by Lindeman, Merenda and Gold [26]. The analysis was performed using the R package "relaimpo" [https://cran.r-project.org/web/packages/relaimpo/relaimpo.pdf]. Since many parameters were measured, variable selection was performed before the relative importance analysis. Ten variables (GLP-1, GLP-2, GIP, insulin AUC, glucose AUC, fasting TG, liver fat, visceral fat, adiponectin and HbA1c) was chosen as variables in common between the three separate analyses with TG plasma AUC, apoB48 AUC and TG chylomicron AUC as response variables respectively. In addition, the top five most correlating variables for each response variable were included. Thus, these five variables varied between each analysis. This design was chosen in order to clarify how GLP-1, GLP-2 and GIP affected postprandial TRL metabolism relative to other common variables and relative to the top correlating parameters for each response variable.

## Results

### Baseline characteristics

**Fig 1:**
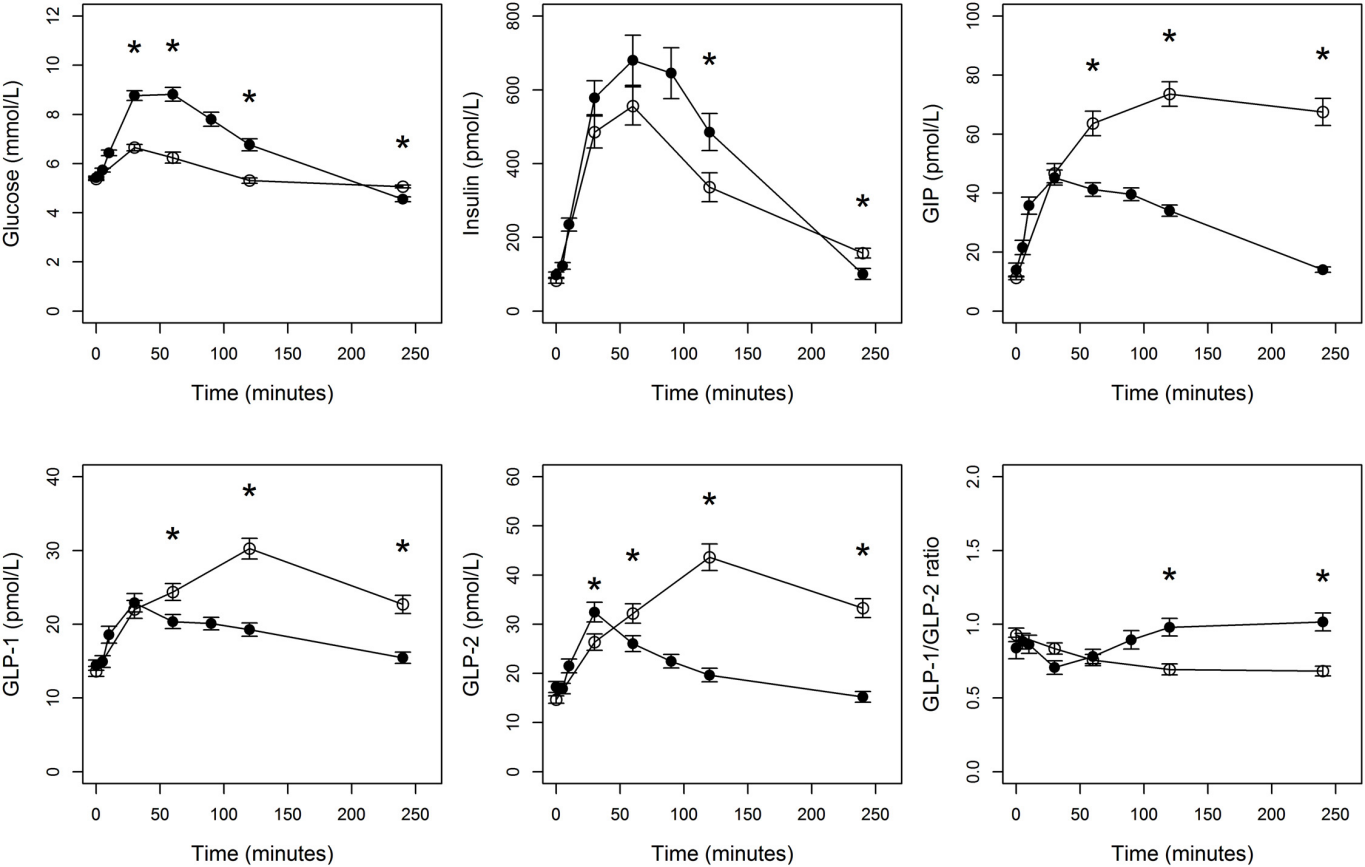
Responses of glucose, insulin, GLP-1, GLP-2 and GIP after fat-rich meal (open circles) and OGTT (black circles). Data show mean ± SEM. Stars indicate significant (p-value < 0.05) difference between fat-rich meal and OGTT for a given time point.

The characteristics of the 65 study subjects are summarized in Table 1. Fasting concentrations of glucose, insulin, GLP-1, GlP-2 and GIP were essentially similar before the OGTT and the test meal, as also illustrated in Fig 1.

**Table 1:** Baseline characteristics. Subject characteristics and fasting concentrations of glucose, insulin, GLP-1, GLP-2 and GIP before fat-rich meal and OGTT. Data are mean ± standard deviation.

	Mean ± SD	Range
Age, years	48.7 ± 10.2	21−65
BMI, kg/m^2^	30.8 ± 3.0	26.5−40.2
Waist, cm	109 ± 8.2	94.5−130.5
Hip, cm	108 ± 8.6	91−137
S-cholesterol, mmol/L	4.96 ± 0.83	3.46−7.05
S-LDL-cholesterol, mmol/L	3.28 ± 0.78	1.94−5.3
S-triglycerides, mmol/L	1.50 ± 0.89	0.34−4.52
S-HDL-cholesterol, mmol/L	1.18 ± 0.32	0.5−2.04
HbA1c, %	5.38 ± 0.32	4.7−6.0
Homa-IR	3.20 ± 0.25	0.46−9.9
Homa-IS, %	121 ± 44.8	32−258
Adiponectin	4.64 ± 2.54	1.4−12.3
***Fat-rich meal***		
P-glucose, mmol/L	5.37 ± 0.45	4.3−6.3
P-insulin, pmol/L	82.2 ± 52.5	11−261
P-GLP-1, pmol/L	13.6 ± 5.41	7−37
P-GLP-2, pmol/L	14.7 ± 6.05	7−46
P-GIP, pmol/lL	11.2 ± 4.89	3−29
***OGTT***		
P-glucose, mmol/L	5.43 ± 0.50	4.5−6.5
P-insulin, pmol/L	98.4 ± 59.0	19−293
P-GLP-1, pmol/l	14.5 ± 5.63	7−31
P-GLP-2, pmol/L	17.2 ± 9.07	4−44
P-GIP, pmol/L	13.9 ± 18.7	3−154

### Relationships between fasting incretins with glycemic and lipid levels in the fasting state

There were no correlations between fasting concentrations of GLP-1, GLP-2 or GIP with BMI, nor with concentrations of fasting glucose, HOMA-IS, fasting plasma TG and apoB48 or fasting TG, apoB48 and apoB100 in chylomicron or VLDL_1_. Fasting GLP-1 but not GLP-2 nor GIP correlated, weakly but significantly, with fasting insulin (*r* = 0.298, *p* = 0.017) and HOMA-IR (*r* = 0.33, *p* = 0.008).

### Glucose, insulin and incretin responses after fat-rich mixed meal and OGTT

First, we explored the impact of the fat-rich meal (56 g fat) and the OGTT on GLP-1, GLP-2 and GIP concentrations as well as on glucose and insulin concentrations (Fig 1). Glucose (AUC 1338 mmol/L×min after fat-rich meal and 1578 mmol/L×min after OGTT, *p*<0.001) and insulin (AUC 79507 mmol/L×min after fat-rich meal and 96911 mmol/L×min after OGTT, *p* = 0.0011) responses were significantly lower after fat-rich meal as compared to OGTT. In contrast, AUC responses for GLP-1, GLP-2 and GIP were significantly (1.4, 1.7 and 2.1 times) higher after the fat-rich meal than after OGTT (Table 2).

**Table 2:** Incretin responses after fat-rich meal and OGTT. Areas under curve (AUC) for glucose, insulin, GLP-1, GLP-2 and GIP after the fat-rich meal and after the OGTT calculated between 0–240 minutes. Data are mean ± standard deviation. The given *p*-value shows differences between fat-rich meal and OGTT.

	Fat-rich meal	OGTT
Glucose (mmol/L × min)	1338 ± 165	1578 ± 280***
Insulin (pmol/L × min)	79507 ± 53934	96911 ± 66490***
GLP-1 (pmol/L × min)	6070 ± 2116	4498 ± 1547***
GLP-2 (pmol/L × min)	8413 ± 3414	4925 ± 2300***
GIP (pmol/L × min)	15125 ± 5968	7375 ± 2946***

*** p<0.001

The times to peak concentrations for glucose (30 min) and insulin (60 min) were similar after fat-rich meal and OGTT and returned to baseline levels at 180 minutes. However, the time of peak concentrations of GLP-1, GLP-2 and GIP during fat-rich meal occurred later, at 120 min, with concentrations remaining elevated at 240 minutes. The AUC responses for GLP-1, GLP-2 and GIP measured after the fat-rich meal correlated with their corresponding AUC responses assessed after the OGTT (S1 Fig). Furthermore, GLP-1 and the GLP-2 AUCs during both the fat-rich meal and the OGTT were significantly correlated, as shown in S2 Fig. GLP-1 and GLP-2 are released in a molar ratio of 1:1 but have different half-lives [27]. To study the relationship between GLP-1 and GLP-2 after the OGTT and after the fat-rich meal we calculated GLP-1/GLP-2 ratios. We observed no significant changes in ratios up to 90 minutes between the two experiments but as GLP-2 values remained elevated at 120 and 240 min after the fat rich meal compared to those after the OGTT, the GLP-1/GLP-2 ratio was significantly lower at 120 and 240 minutes after the fat rich meal (Fig 1).

### Postprandial responses of lipids and lipoproteins after fat rich mixed meal

After the test meal, plasma TG and apoB48 levels, as well as TG, apoB48 and apoB100 in chylomicron and VLDL_1,_ increased significantly from fasting concentrations (S1 Table). S3 Fig displays the curves for plasma total TG and apoB48. The peak concentrations for both serum TG and apoB48 were observed at the 240 min time point and thereafter started to decline. Notably, plasma apoB48 rose more rapidly than TG levels, with an early peak at 120 min which was not evident in the plasma TG curve, and remained elevated up to 240 min. The maximum concentrations of TG and apoB48 were reached in chylomicrons and VLDL_1_ fractions at 240 minutes (data not shown).

### Relationships between incretin AUCs *vs*. TRLs during the fat-rich meal

Weak positive correlations were observed between glucose and insulin AUC after the fat-rich meal and triglycerides and apoB48 in plasma, chylomicrons and VLDL_1_ (S2 Table). Fasting adiponectin correlated negatively with triglycerides in plasma, chylomicrons and VLDL_1_. We observed no relationships between adiponectin and GLP-1, GLP-2 or GIP.

**Fig 2:**
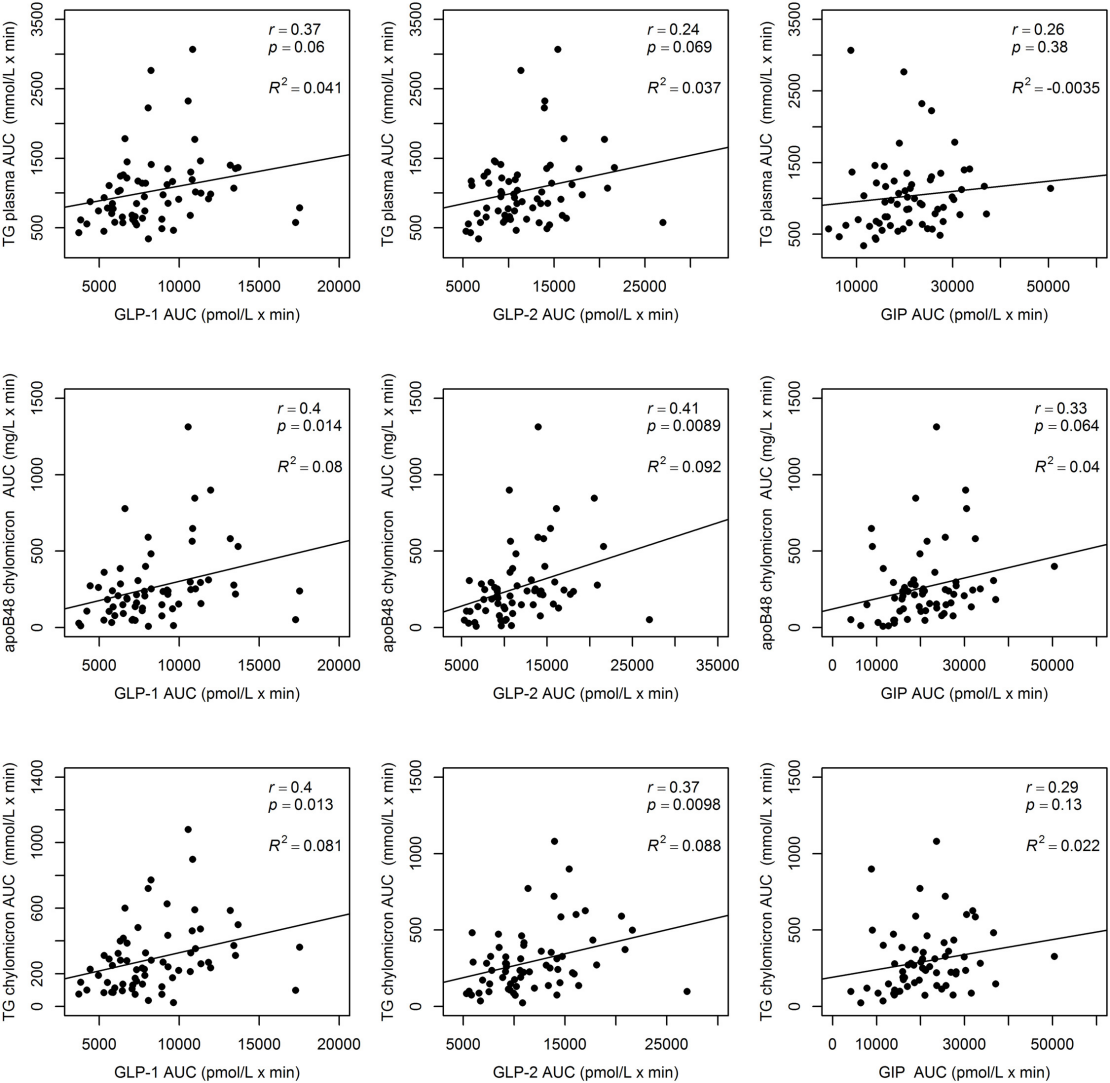
Correlation between incretins and postprandial lipid responses. Correlations between areas under curve (AUC) for GLP-1, GLP-2 and GIP with plasma TG, apoB48 in the chylomicron fraction, and triglycerides in the chylomicron fraction after the fat-rich meal.

**Fig 3:**
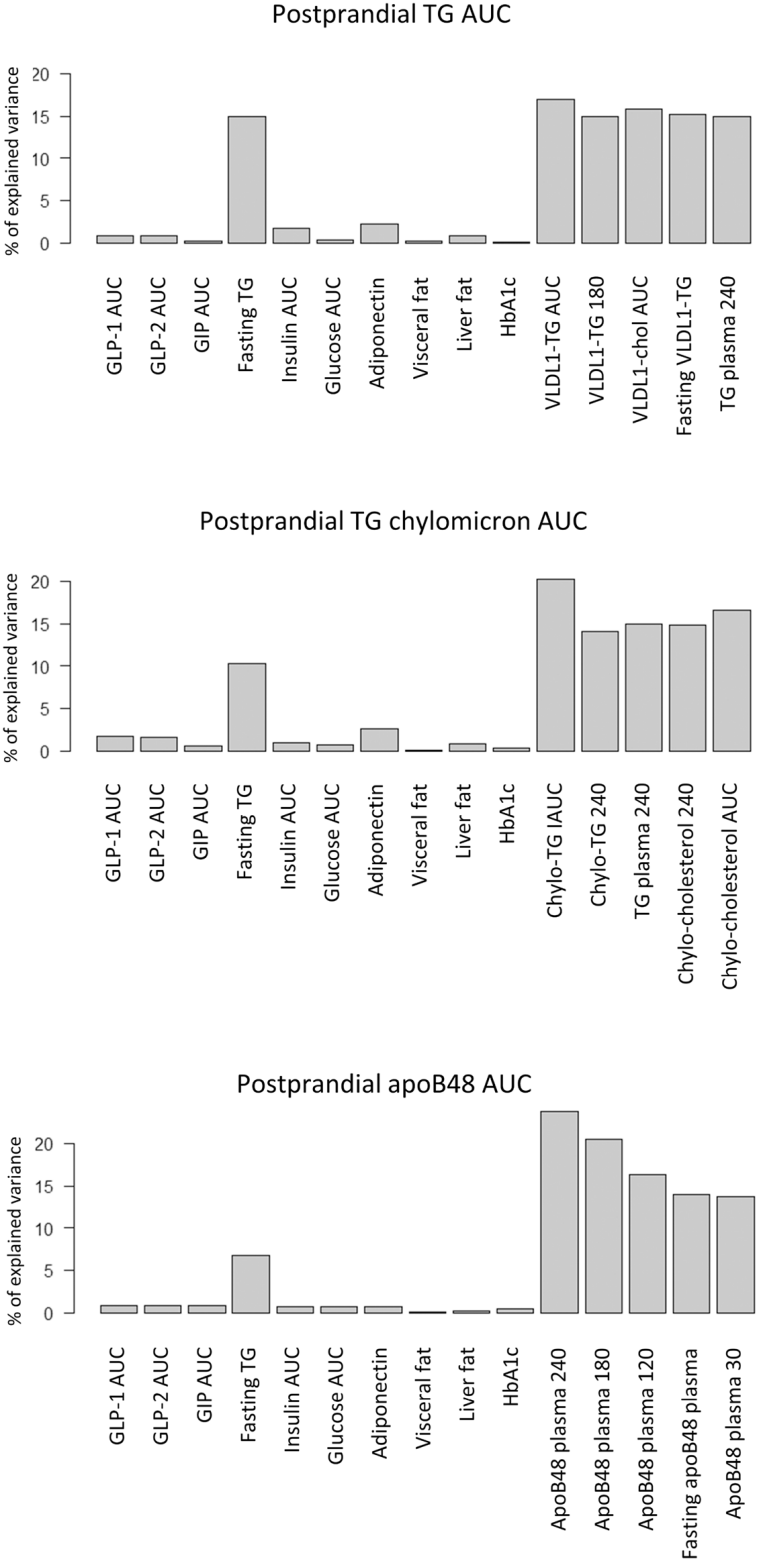
Relative importance analysis. Contribution of each variable for explaining (A) the plasma TG AUC (B) the TG-chylomicron AUC and (C) the apoB48 AUC after the fat-rich meal. For every plot 15 variables are shown. Ten variables including GLP-1, GLP-2 and GIP (to the left in each plot) are shared between (A), (B) and (C). Five variables to the right hand side in the graphs vary between (A), (B) and (C). These five were chosen as they correlate most strongly with the response variable in question and thus provide a relative importance reference to the other ten variables.

To study the relationship between incretin and postprandial TRL responses during the fat rich meal, we first calculated the Spearman correlations between GLP-1, GLP-2, GIP and TG, apoB48 and apoB100 AUC responses in plasma, chylomicrons and VLDL_1_ (Fig 2). Significant, positive correlations were observed between GLP-1 as well as GLP-2 AUCs and GIP AUCs with AUCs for TG and apoB48 in chylomicrons, whereas their relationships with plasma TG and apoB48 levels were less consistent. Overall however, these correlations were moderate to weak and as shown in Fig 2, the r-squared values for these correlations were close to zero. Furthermore, the results from the relative importance analysis (Fig 3) shows that GLP-1, GLP-2 and GIP contributed very little to explaining the variance in TG plasma AUC, apoB48 AUC and TG chylomicron AUC. Insulin AUC, glucose AUC, adiponectin, liver fat, visceral fat and HbA1c did not either display large individual contributions to explaining the variance in postprandial TG excursion. The individual largest explanatory variable for postprandial TG excursion among the commonly measured clinical risk factors was fasting triglycerides.

## Discussion

Here we report endogenous responses of GLP-1, GLP-2 and GIP after both fat-rich meal and OGTT in healthy obese males and to show that the responses of these three metabolic gut hormones were highly correlated with each other. We also show that the responses of these gut hormones were significantly higher and more prolonged after the fat-rich meal than in response to the OGTT, although postprandial plasma glucose and insulin levels remained lower than during the OGTT. After the fat-rich meal, concentrations of GLP-1, GLP-2 and GIP continued to increase up to 240 min compared to early peaks observed at 30 minutes during the OGTT. Notably, the time pattern of glucose and insulin responses after the fat-rich meal mimicked the time pattern after the OGTT, but the responses of both glucose and insulin were as expected higher after the OGTT. The responses of both TG and apoB48 in chylomicrons after the fat-rich meal correlated significantly positively with both GLP-1 and GLP-2 responses despite the fact that GLP-1 and GLP-2 are reported to have divergent effect on intestinal lipid metabolism [28]. However, the correlations were weak, as the low r-squared values indicate. Large variation in postprandial TRL AUCs was observed between subjects with similar GLP-1 and GLP-2 responses. In addition, the relative importance analysis revealed that GLP-1, GLP-2 and GIP AUCs did not explain much of the variation in TRL AUCs. Our data thus provide no evidence for a seminal role of endogenously produced GLP-1, GLP-2 or GIP on postprandial metabolism of TRL particles but highlight the predictive role of fasting triglycerides as previously documented [12].

Both carbohydrate and fat ingestion are physiological stimuli for secretion of gastrointestinal hormones [29, 30]. We utilized OGTT as a reference test for the fat-rich meal as most of the current knowledge on incretin responses is derived from OGTT performed in healthy subjects as well as in metabolic diseases. The responses of GLP-1 and GIP after an oral fat challenge have been reported to be higher than after an intravenous administration at matched plasma TG concentrations, suggesting an analogous response of the incretin hormones after an oral lipid load as seen after OGTT [21]. In addition, Nauck *et al* reported a strong correlation between GLP-1 and GIP responses of OGTT [31]. In the present study, both GLP-1 and GIP in response to a fat-rich meal and OGTT increased promptly at 30 minutes, but peaked much later at 120 minutes after the fat-rich meal compared to early peaks observed after the OGTT. The overall responses of GLP-1, GLP-2 and GIP after the fat rich meal were markedly higher than after the oral glucose challenge. In fact, the response of GIP was about 2-fold higher after the fat-rich meal than after the OGTT, and the values remained elevated up to 240 minutes, in line with Vollmer *et al* who reported that GIP response to a meal test containing 820 kcal (353 kcal from fat) was markedly higher and prolonged compared to OGTT [32]. These differences may be due the differences in gastric emptying of the served nutrients that is known to modify the release of incretin hormones. Indeed, the two tests in our study differed markedly in size, consistency, and fat content. This likely affected the gastric emptying and thus the slope of the curves in Fig 1.

Fat, protein and carbohydrates stimulate the release of GLP-1 and GIP in healthy humans. We opted to use a fat-rich meal containing all three nutrients as this represents a more physiologically relevant meal than serving pure olive oil, a protein mix [33], lipid emulsions [21] or constant intraduodenal feeding (18). As the fat-rich meal contained a significant amount of protein (40 g), we cannot exclude that part of the sustained responses of GLP-1 and GIP could be linked to protein ingestion [34]. In addition the high energy (927 kcal for the fat-rich meal compared to 300 kcal for the OGTT) and high fat content of the meal may have influenced the absorption of nutrients and thus also the release of incretin hormones.

A wealth of evidence from both human and animal studies demonstrate that incretin-based therapies not only improve glycemic control, but also improve the lipid profiles in patients with T2D [13, 16, 29, 35–37]. Infusion of synthetic GLP-1 in pharmacological doses has been shown to reduce intestinal lymph flow, handling and absorption of dietary fat and intestinal secretion of apoB48 resulting in reduction of postprandial triglycerides in rats and in human [38]. Data from recent intestinal lipoprotein kinetic studies suggest that the action of GLP-1 on the production and release of intestinal TRL particles is inhibitory, leading to the lowering of postprandial triglyceride excursions [16, 37, 39]. The elegant design of the lipoprotein kinetic studies allows us to conclude that exenatide, a GLP-1 receptor agonist, has a direct effect on intestinal lipoprotein production beyond its actions on gastric emptying and nutrient absorption [28]. Likewise, the suppression of postprandial lipid and apoB48 responses to fat feeding has been confirmed in animal studies [13]. Our observation that GLP-1 response to fat-rich meal explained only a minor part in the variation of postprandial responses of triglycerides or apoB48 in chylomicrons seems to contradict a primary role for GLP-1 in the regulation of chylomicron release.

GLP-2 is secreted from intestinal L cells together with GLP-1 in a 1:1 molar ratio in response to nutrient intake [30]. GLP-2 has trophic actions in intestine and its major effects are to enhance intestinal cell growth, intestinal blood flow as well as increase absorption of major macronutrients [40]. It should be recognized that GLP-2R is not expressed in enterocytes. Thus the potential actions of GLP-2 on intestinal lipid metabolism have remained mostly unnoticed. However, a recent review highlighted that GLP-2 may have divergent effects from GLP-1 on intestinal lipid metabolism [28]. Intravenous infusion of GLP-2 during a solid meal stimulated postprandial responses of triglyceride compared to placebo in healthy humans most likely due to enhanced lipid absorption [41]. When GLP-2 was injected acutely subcutaneously, it increased the intestinal secretion of apoB48 and chylomicrons [42]. In this elegant study, Dash et al. demonstrated that acute injection of GLP-2 enhanced release of pre-synthesized apoB48 stored in the gut. In our study, we observed very rapid increases of both TRL triglycerides and apoB48 after the fat-rich meal and the chylomicron triglyceride and apoB48 AUC correlated significantly but weakly with the responses of GLP-2. In Syrian hamster and mice, GLP-2 has been reported to enhance fat absorption and intestinal release of TRLs and increase apoB48 responses to fat feeding [17, 43, 44]. Our data from the relative importance analyses suggest that GLP-2, like GLP-1, can provide only limited explanatory power for the variation of chylomicron responses to fat-rich meal. The data does not exclude that GLP-1 and GLP-2 may modify intestinal lipid metabolism, however it indicates that exogenously administered pharmacological doses of GLP-1 and GLP-2 analogues may have diverse action on some pathways compared with endogenously released hormones leaving the contribution of GLP-1 and GLP-2 on intestinal chylomicron metabolism still open.

Dietary lipids are either assembled into chylomicrons and secreted into lymphatic vessels, or stored in the intestinal cells as lipid droplets for the storage of fat to be mobilized later [45]. Interestingly, we observed a rapid increase of plasma apoB48 in response to fat-rich meal, but the response of plasma triglycerides was delayed and less robust at the early time point after the fat-rich meal. This early release of apoB48 agrees well with the observation by Dash et al. that GLP-2 injection increased TRL triglyceride and apoB48 levels already at 30 minutes [42]. The authors concluded that GLP-2 has a special effect on the mobilization of prestored apoB48 from enterocytes [17, 28, 42]. Recently the preferential release of triglycerides from intestinal storage pool by GLP-2 injection intraperitoneally was confirmed in Syrian golden hamster [44]. The results are consistent with the concept of the “second meal effect” that is considered to reflect the utilization of storage pool lipids derived from previous meals for chylomicron release [46, 47]. This concept becomes important when we face successive fat rich meals in our daily food consumption. An important question is whether the amount of ingested fat is a determinant of intestinal fat stores and postprandial lipemia leading to the accumulation of thermogenic remnants in the circulation.

Lipid digestion has been shown to release long chain fatty acids (LCFA) that are essential for lipid sensing, not only in the gut, but also in the tongue taste buds and in the brain [48]. This capacity for fat taste has been proposed to modify intestinal lipid handling via rapid incretin secretion after meal ingestion. The fact that both GLP-1 and GLP-2 concentrations were increased at 30 and 60 minutes after the fat-rich meal, preceding the increase of apoB48 at 60 and 120 minutes and TG at 120 minutes, might indicate that these responses of GLP-1 and GLP-2 relate to fat tasting and may elicit the release of pre-stored apoB48 and chylomicrons from lipid droplets in enterocytes.

The strength of our study is that all three metabolic hormones were measured during both a fat-rich meal as well as a standard OGTT. This, together with a comprehensive analysis of TRL particles, allowed us to follow the dynamics of both chylomicrons from the intestine and liver-derived VLDL_1_ after a fat-rich meal. The study has some limitations, it is observational and the nutritional challenges were given as single loads. We did not have a metabolically healthy lean cohort for comparison, meaning that the results cannot be extrapolated to different cohorts. Additionally, the conclusions are based primarily on advanced statistical analysis, so appropriate intervention studies are needed to dissect out the actions of the individual hormones on lipid metabolism. Thus, we cannot unveil the molecular mechanisms behind the responses of GLP-1, GLP-2 and GIP to fat ingestion. In conclusion, our data do not support a decisive role for the three intestinal hormones in the regulation of postprandial lipid metabolism after a fat-rich meal. It is important to emphasize that the endogenous responses of GLP-1, GLP-2 and GIP reported in this study, do not have to correspond to responses after pharmacological intervention targeting GLP-1 or GLP-2.

## Supporting Information

S1 Fig Postprandial responses of incretins. Correlations for each incretin response between the fat-rich meal and the OGTT. (A) GLP-1 (r = 0.73; *p*<0.001), (B) GLP-2 (r = 0.46; *p*<0.001) and (C) GIP (r = 0.69; *p*<0.001) measured after the fat-rich meal correlated significantly with the respective measurement after the OGTT.
(PDF)

S2 Fig Postprandial correlations of GLP-1 and GLP-2. Correlations between GLP-1 and GLP-2 AUC (A) after OGTT (*r* = 0.52; *p*<0.001) and (B) after fat-rich meal (*r* = 0.56; *p*<0.001).
(PDF)

S3 Fig Plasma TG and apoB48 concentrations after the fat-rich meal. A more rapid increase in apoB48 compared to TG is evident. Data show mean ± SEM.
(PDF)

S1 Table Postprandial responses after a fat-rich meal. After the fat-rich meal, significant changes in TRL parameters were observed. Stars indicate significant changes compared to baseline. *P<0.05; **P<0.01; ***<0.001.
(PDF)

S2 Table Postprandial responses after fat-rich meal. Spearman correlation coefficients between postprandial TRL AUCs and Insulin AUC, Glucose AUC, fasting adiponectin, HOMA-IR and HOMA-IS. *P<0.05; **P<0.01; ***<0.001.
(PDF)

## Abbreviations

apo: apolipoprotein
AUC: area under curve
DPP-4: dipeptidyl peptidase-4
GLP-1: glucagon-like peptide-1
GLP-2: glucagon-like peptide-2
GIP: glucose-dependent insulinotropic polypeptide
HDL: high density lipoproteins
iAUC: incremental area under curve
LDL: low density lipoproteins
T2D: type 2 diabetes
TRL: triglyceride-rich lipoprotein
TG: triglyceride
VLDL: very low-density lipoprotein
