# Supplementary figures and images for: Minor Contribution of Endogenous GLP-1 and GLP-2 to Postprandial Lipemia in Obese Men
Source: PLoS One. 2016 Jan 11;11(1):e0145890. doi: 10.1371/journal.pone.0145890 (PMC4709062; doi:10.1371/journal.pone.0145890)

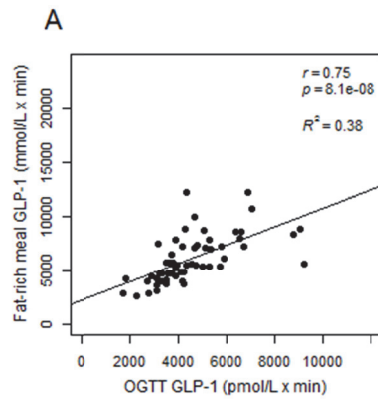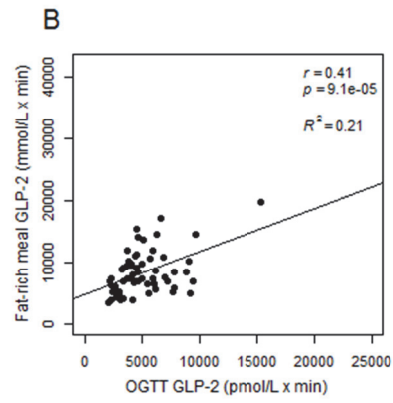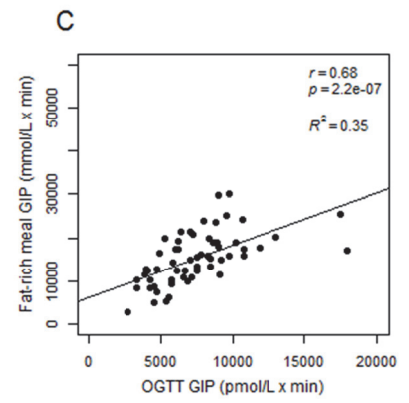

Supplement: S1 Fig — Correlations for each incretin response between the fat-rich meal and the OGTT. (A) GLP-1 (r = 0.73; p<0.001), (B) GLP-2 (r = 0.46; p<0.001) and (C) GIP (r = 0.69; p<0.001) measured after the fat-rich meal correlated significantly with the respective measurement after the OGTT. (PDF) [file pone.0145890.s001.pdf]

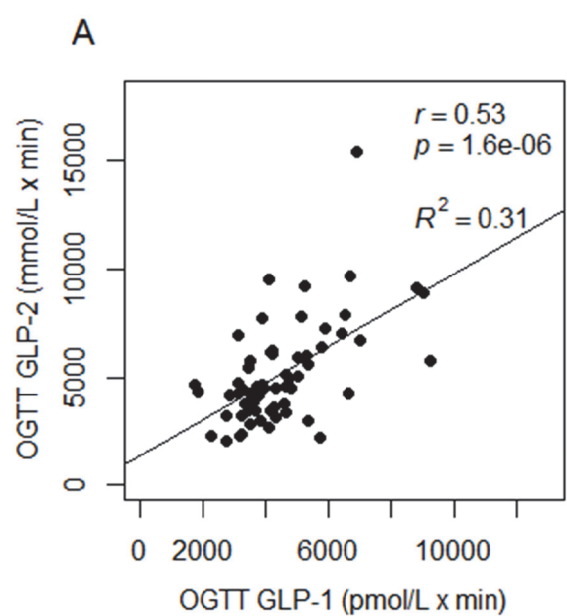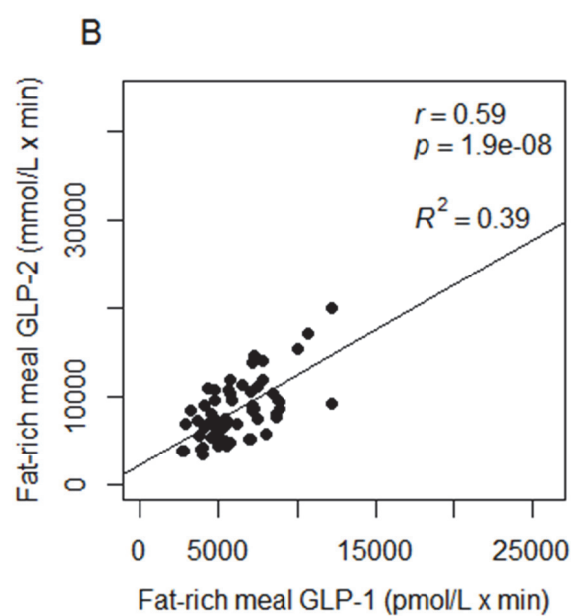

Supplement: S2 Fig — Correlations between GLP-1 and GLP-2 AUC (A) after OGTT (r = 0.52; p<0.001) and (B) after fat-rich meal (r = 0.56; p<0.001). (PDF) [file pone.0145890.s002.pdf]

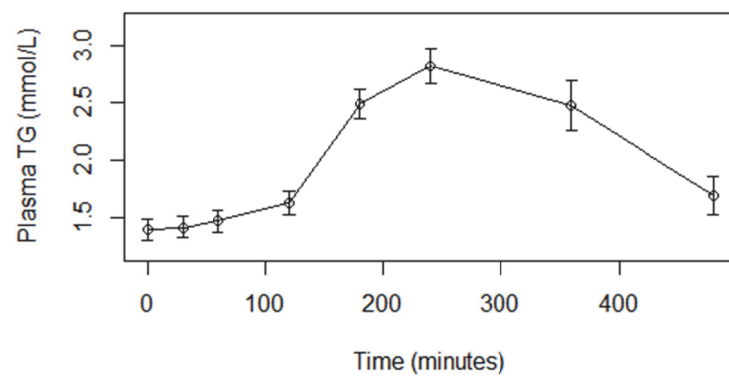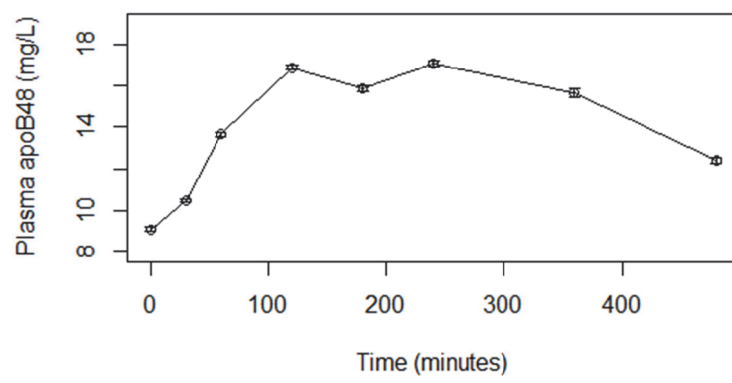

Supplement: S3 Fig — A more rapid increase in apoB48 compared to TG is evident. Data show mean ± SEM. (PDF) [file pone.0145890.s003.pdf]
