# Supplementary material for: Minor Contribution of Endogenous GLP-1 and GLP-2 to Postprandial Lipemia in Obese Men
Source: PLoS One. 2016 Jan 11;11(1):e0145890. doi: 10.1371/journal.pone.0145890 (PMC4709062; doi:10.1371/journal.pone.0145890)
Supplement: S1 Table — After the fat-rich meal, significant changes in TRL parameters were observed. Stars indicate significant changes compared to baseline. *P<0.05; **P<0.01; ***<0.001. (PDF) [file pone.0145890.s004.pdf]

	Baseline mean ± SD	180 min mean ± SD	240 min mean ± SD	360 mean ± SD	480 min mean ± SD
TG plasma (mmol/L)	1.39 ± 0.78	2.49 ± 1.03 **	2.82 ± 1.20 **	2.48 ± 1.70 **	1.69 ± 1.29 *
TG Chylo (mmol/L)	0.11 ± 0.13	0.78 ± 0.51 **	0.96 ± 0.63 **	0.70 ± 0.85 **	0.31 ± 0.45 **
TG VLDL1 (mmol/L)	0.57 ± 0.47	0.86 ± 0.48 **	0.94 ± 0.53 **	0.87 ± 0.63 **	0.59 ± 0.63
apoB48 Chylo (mg/L)	0.05 ± 0.10	0.67 ± 0.56 **	0.87 ± 0.75 **	0.68 ± 0.87 **	0.3 ± 0.54 **
apoB48 VLDL1 (mg/L)	0.58 ± 0.56	1.67 ± 0.80 **	1.79 ± 0.90 **	1.62 ± 1.16 **	1.04 ± 1.18 **
apoB100 Chylo (mg/L)	0.72 ± 1.15	1.25 ± 1.35 **	1.44 ± 1.48 **	1.47 ± 1.87 **	0.97 ± 1.78 *
apoB100 VLDL1 (mg/L)	26.8 ± 26.1	37.0 ± 30.9 **	40.5 ± 36.1 **	35.9 ± 28.5 **	25.4 ± 25.9
apoB48 plasma (mg/L)	9.10 ± 4.41	15.9 ± 5.13 **	17.1 ± 6.58 **	15.6 ± 7.90 **	12.4 ± 7.36 **
