# Supplementary material for: Minor Contribution of Endogenous GLP-1 and GLP-2 to Postprandial Lipemia in Obese Men
Source: PLoS One. 2016 Jan 11;11(1):e0145890. doi: 10.1371/journal.pone.0145890 (PMC4709062; doi:10.1371/journal.pone.0145890)
Supplement: S2 Table — Spearman correlation coefficients between postprandial TRL AUCs and Insulin AUC, Glucose AUC, fasting adiponectin, HOMA-IR and HOMA-IS. *P<0.05; **P<0.01; ***<0.001. (PDF) [file pone.0145890.s005.pdf]

Fat-rich meal TRL AUCs	Insulin AUC	Glucose AUC	Adiponectin	HOMA-IR	HOMA-IS
TG Plasma(mmol/L × min)	0.32 *	0.29*	-0.39 **	0.22 *	0.12
TG Chylo (mmol/L × min)	0.27	0.26 *	-0.42 **	0.10	0.01
TG VLDL1 (mmol/L × min)	0.30 *	0.28	-0.42 **	0.22 *	0.11
ApoB48 Plasma (mg/L x min)	0.19	0.21	-0.14	0.30	0.14
ApoB48 Chylo (mg/L × min)	0.18	0.10	-0.21	0.10	0.02
ApoB48 VLDL1 (mg/L × min)	0.33 *	0.27	-0.29 **	0.28 *	0.14 *
